# Neurological Involvement in Pediatric Patients with Acute Leukemia: A Retrospective Cohort

**DOI:** 10.3390/children9091268

**Published:** 2022-08-23

**Authors:** Diana Alejandra Cruz-Chávez, Brian Javier López-Pérez, Elsa Solórzano-Gómez, José Antonio Venta-Sobero, Luz Victoria Flores-Villegas, Christian Gabriel Toledo-Lozano, Gabriela Vianney Castro-Loza, Roberto Sandoval-Pacheco, Andrea Torres-Vallejo, Karen Sharlot Faisury Marmol-Realpe, Yazmín Evelyn Flores-Jurado, Cristal Lucero Hernández-Soriano, Sofía Lizeth Alcaraz-Estrada, Paul Mondragón-Terán, Juan Antonio Suárez-Cuenca, Ramón Mauricio Coral-Vázquez, Silvia Garcia

**Affiliations:** 1 Department of Pediatric Neurology, Centro Médico Nacional “20 de Noviembre”, Instituto de Seguridad y Servicios Sociales para los Trabajadores del Estado, Mexico City 03229, Mexico; 2 Department of Pediatric Hematology, Centro Médico Nacional “20 de Noviembre”, Instituto de Seguridad y Servicios Sociales para los Trabajadores del Estado, Mexico City 03229, Mexico; 3 Department of Clinical Research, Centro Médico Nacional “20 de Noviembre”, Instituto de Seguridad y Servicios Sociales para los Trabajadores del Estado, Mexico City 03229, Mexico; 4 Department of Undergraduate Research, Hospital Militar de Especialidades de la Mujer y Neonatología, Mexico City 11200, Mexico; 5 Department of Pediatric Endocrinology, Instituto Nacional de Pediatría, Mexico City 03700, Mexico; 6 Department of Genomic Medicine, Centro Médico Nacional “20 de Noviembre”, Instituto de Seguridad y Servicios Sociales para los Trabajadores del Estado, Mexico City 03229, Mexico; 7 Department of Teaching and Research, Centro Médico Nacional “20 de Noviembre”, Instituto de Seguridad y Servicios Sociales para los Trabajadores del Estado, Mexico City 03229, Mexico; 8 Postgraduate Section, Escuela Superior de Medicina, Instituto Politécnico Nacional, Mexico City 11340, Mexico; 9 Department of Neuroscience, Centro Médico Nacional “20 de Noviembre”, Instituto de Seguridad y Servicios Sociales para los Trabajadores del Estado, Mexico City 03229, Mexico

**Keywords:** acute leukemia, neurological manifestations, children

## Abstract

Acute leukemia (AL) is an important cause of morbidity and mortality in children, and neurological manifestations (NM) are frequent. The objective of this study was to analyze neurological manifestations in children with acute leukemia from cases attended in the last five years at the Centro Médico Nacional “20 de Noviembre”. Methods: Conducting a retrospective and analytical study from 1 January 2015 to 31 December 2020 in children with AL classified according to sex, age range and AL type. Participants were grouped according the presence of NM. Results: We analyzed 607 patients: 54.85% boys and 44.14% girls, with a mean age of 7.27 ± 4.54 years. When comparing groups, the NM group was significantly older (*p* = 0.01), and the highest prevalence was between 6 and 12 years old. ALL was predominant over the other lineages (*p* ≤ 0.01). The most frequent NM was CNS infiltration, seizures, headache and neuropathy. Death outcomes occurred in 18.7% of children with AML, 11.8% with ALL and 50% with MPAL (*p* ≤ 0.002). The NM group was associated with higher mortality during a follow-up time of 77.9 ± 49 months (44.4% vs. 8.9% deaths, NM vs. non-NM, respectively; OR = 3.3; 95% CI 2.4 to 4.6; *p* ≤ 0.0001). Conclusions: ALL was the most prevalent leukemia type. CNS infiltration, seizures, headache, neuropathy and PRES were the most frequent symptoms in the NM group. NM was associated with a higher mortality rate.

## 1. Introduction

Acute Leukemia (AL) is a malignant disease where the exact cause is unknown and for which its etiologies probably involve multiple factors, such as ionizing radiation, chemicals, drugs, infections, genetic factors and chromosomal abnormalities [1]. AL occurs in one-third of children with cancer, and it is the most common type of cancer at this age. Fortunately, the survival rate is around 90% [1,2,3]; however, AL is still the leading cause of death by a single disease at this age [4,5]. Clinical manifestations occur as a result of bone marrow (BM) failure and include diverse and nonspecific symptoms such as bleeding, petechiae, purpura, fatigue, anorexia, malaise, bone pain and pallor [1].

In Mexico, the Ministry of Health has reported an annual incidence of 2500 to 3000 cases, in which males were slightly more prevalent (56%). The mortality rate is between 4.35 and 6.88/100,000 children between 0 and 19 years old [6].

Leukemia survival has improved in recent decades, especially in acute lymphoblastic leukemia (ALL), as a result of its molecular characterization as well as the use of therapies adapted to the risk and phase of disease and optimized combined chemotherapy schemes, including prednisone or dexamethasone, vincristine, asparaginase, anthracycline, mercaptopurine, cyclophosphamide and cytarabine, leading to 90% full remission rates in pediatric patients [7,8].

The more effective Central Nervous System (CNS) prophylaxis and enhanced chemotherapy after induction and better supportive care are particularly relevant; however, the increased survival rates resulted in greater neurotoxicity [1,9]. The condition of AL in childhood significantly affects the quality of life at a stage in which the acquisition of motor and cognitive skills is essential [10]. Fortunately, therapies for Acute Myeloid Leukemia (AML), Mixed-Phenotipe Acute Leukemia (MPAL) [11] and ALL have increased their effectiveness [11,12]. CNS and Peripheral Nervous System (PNS) involvement in AL has been associated with Leukemic infiltration, chemotherapy, radiotherapy, infection, hemorrhage, cerebrovascular lesions, metabolic/hydroelectrolytic imbalance, syndromes of the inappropriate secretion of antidiuretic hormone or nutritional deficiencies [1,13,14,15,16,17], which are all mostly symptoms occurring in the first two months of therapy [18]. Specific neurological manifestations vary according to the structures, neuron component and neuroanatomical location involved [19]. Atypical clinical manifestations such as headache, loss of balance, fainting, mood swings, seizures, nausea/vomiting and papilledema are often present. Cranial nerve involvement is less common and can cause diplopia, facial numbness, hearing loss, blindness and swallowing difficulties [12].

The aim of this study was to describe the NM of pediatric patients diagnosed with AL and to identify potentially associated demographic and clinical characteristics, risk factors and clinical outcomes.

## 2. Materials and Methods

A retrospective, longitudinal, analytical study involving 607 pediatric patients with AL diagnosis, who attended at the Hematology Department from Centro Médico Nacional “20 de Noviembre” between 1 January 2015 and 31 December 2020, was performed. Inclusion criteria included age younger than 18 y-o, a diagnosis of AL made by a board-certified hematologist with expertise in childhood leukemias and using the criteria proposed by the Franco-British Cooperative Group [10,12]. Patients were excluded if there were incomplete data in the clinical record or a non-clear identification of any clinical manifestation. Cases were also eliminated if the initial hematological diagnosis was modified during the study’s follow-up period.

Data collection was obtained from clinical records, and variables included Demographic and clinical data (age, sex, type of leukemia, age of onset of AL, clinical manifestations, hematological drug- and radio-therapies used and mortality outcome). Clinical manifestations were collected; in particular, NMs were defined as any CNS and/or PNS symptom, signs or laboratory findings that occurred during the time period considered in the study. NMs were identified by a board-certified pediatric neurologist.

Chemotherapy schemes were based on recommendations from Institutional Diagnostic and Therapeutic Guidelines from the Pediatric Hematology Department of the Cen-tro Médico Nacional “20 de Noviembre “, Mexico City; in accordance with international chemotherapy schemes. Briefly, ALL chemotherapy included the St. Jude XV protocol (based on schemes including steroids, cytarabine, vincristine, daunorubicin, L-asparaginase, cyclophosphamide, 6-mercaptopurine, Ara C, folinic acid and methotrexate). Other schemes used was LALRA (Intermediate and High Risk Relapsed Acute Lymphoblastic Leukemia), based on schemes from the Memorial Sloan Kettering Hospital (including daunorubicin, cytarabine and methotrexate), as well as schemes for ALL with Isolated Relapse to CNS (POG), including high doses of methotrexate and cytarabine, intra-thecal chemotherapy and hydrocortisone. On the other hand, AML was treated with a scheme based on mercaptopurine, cytarabine, steroid, dexrazoxane, idarubicin, etoposide and mithoxantrone, whereas MPAL chemotherapy may include adjusted schemes according to cell’s lineage predominance.

The study population was divided into two groups (group 1, patients with NM; group 2, patients without NM) for comparative purposes.

Statistical analysis was performed with IBM SPSS version 23 package. Descriptive and inferential analyses were performed according to the type of variable, using Chi-square, Student’s T-test (mean ± standard deviation), Odds Ratio, product-limit estimator (survival analysis) and proportional hazards regression at 95% confidence intervals. Statistical significance was considered if *p* < 0.05.

## 3. Results

The study population comprised 607 children with AL in which 54% were males with a mean age of 7 years old, whereas 61.4% were younger than 10 years old. 

In general, NM as the first clinical manifestation occurred in 17.4% of the study population. This subgroup of children was characterized at 9.7 ± 4.5 years old, and when comparing groups, patients with NM were significantly older (*p* = 0.01); the highest prevalence occurred at the range of 6 to 12 years old. The mean follow-up time of participants was 47.9 + 38.2 months. Other sociodemographic data are shown in Table 1.

ALL predominates over other lineages, followed by AML and MPAL. Significant differences were found regarding sex, although no case with MPAL was reported in girls. 

The age at diagnosis for ALL was 6.3 ± 3.9 years old, which was significantly younger than AML presenting at 12.3 ± 4.3 years old (*p* < 0.0001). See Table 2. 

Regarding the type of NM, CNS infiltration was observed in 26.4% of the cases; all of them were identified during the re-induction phase. On the other hand, 73.6% showed neurologic deficits or other NM symptoms, and the most frequent includes seizures at 26.4% followed by headache 18.9%, neuropathy 11.3% and others. Most symptoms appeared during induction and re-induction phases. The phase of onset of the other NM is also shown for comparison. Seizures mostly appeared during the chemotherapy induction phase, and the coexistence with CT-toxicity cases was also shown. Chemotherapy schemes that were most used in patients with NM were St Jude XV (61%) and LALRA (30%). (See Table 3). Other potential etiologies in relation with NM that were not clearly established were vascular lesions (4.7%), systemic infections (3.8%), metabolic disorders (3.8%) and other miscellaneous conditions (17%). 

Since some symptoms transformed along different chemotherapy phases, the final neurological diagnosis was also considered. CNS infiltration was the most prevalent followed by epilepsy and primary headache. The other diagnoses are shown in Table 4.

Regarding the time to NM onset, we found a mean of 18.9 ± 22.6 months, while the time from NM to death was 13.0 ± 19.5 months. In the subgroup with ALL, the time to NM on-set was 18.4 + 21.7 months, whereas other linages (AML and MPAL) showed a mean of 22.3 + 27.7 months. Finally, the time from NM to death for ALL was 14.2 + 20.9 and 8.8 + 13.3 for ALL compared with other linages (AML and MPAL), respectively (*p* = 0.525).

**Figure 1:**
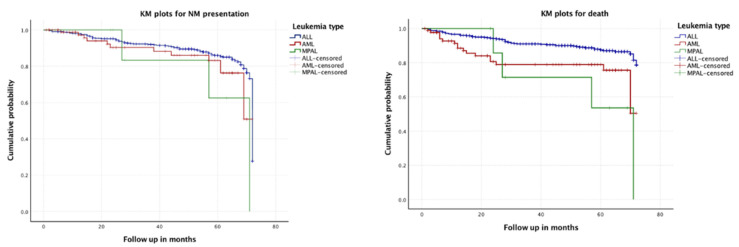
Kaplan–Meier analysis. Presentation of NM (**left panel**) and mortality (**right panel**) during follow-up according to leukemia types. Cases with ALL are shown in blue, with AML in red and with MPAL in green. Abbreviations: KM, Kaplan–Meier; NM, neurological manifestations; ALL, acute lymphoblastic leukemia; AML, acute myeloid leukemia, MPAL, mixed-phenotype acute leukemia.

Finally, mortal outcomes occurred in 18.7% of children with AML, 11.8% with ALL died and 50% with MPAL (*p* ≤ 0.002), whereas an older age at diagnosis was within the group of ALL (8.59 ± 4.85 vs. 7.06 ± 4.46, *p* ≤ 0.005). In time-to-event analyses according to leukemia types, for NMs, no statistically significant differences were found (*p* = 0.424), while they were found for mortality (*p* < 0.001). See Figure 1.

**Figure 2:**
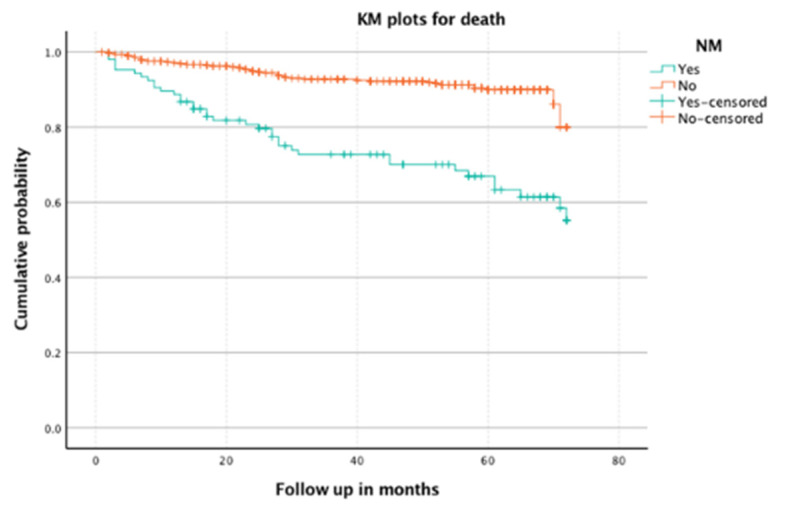
Kaplan–Meier analysis. Mortality associated with NM. Abbreviations: KM, Kaplan Meier; NM, neurological manifestations.

Specifically, the NM-group was associated with higher mortality during a follow-up time (44.4% vs. 8.9% deaths, NM vs. non-NM, respectively; HR = 3.83; 95% CI 95% 2.5 to 5.96; *p* ≤ 0.0001, Figure 2), whereas 36.7% and 42.6% died during the first and second years of follow up. No differences were observed regarding sex.

## 4. Discussion

The aim of this study was to characterize NM in children with AL. In general, gender distribution in our study population was 1.21/1 for a boy/girl ratio, which is comparable to other studies of hematology malignancies [20]. ALL was more frequently diagnosed at younger age than other leukemias, and this may be relevant, since this type requires a more aggressive treatment and is associated with greater morbidity [4,21].

The presence of NM is frequent during the course of AL not only at diagnosis as but also during relapses [22,23], which is in accordance to the results observed in the study population. In particular, cases with NM tended to be older. This is consistent with literature reports [4,24,25]. Age, but not sex, significantly associated with NM, where the group within the range of 6 to 12 years old was more affected. Similarly, previous studies [1,4] reported a higher frequency of NM at this age, and this may be explained by a coincidence with the age of presentation for ALL, which was the most prevalent type of leukemia in our study as well as in other reports [20,21].

According previous studies, up to 29% of cases with AL presenting NM [13,23] were higher relative to the findings of this study. The onset time of NM was shorter for ALL compared with other lineages [13,18].

CNS infiltration was the most frequent neurological event (55%), which was higher than that reported in the literature (3% to 40%) [1,20,26,27], and this is possibly due to the inclusion of children with NM as well as asymptomatic cases with neurological damage; likewise, survival increased over recent years [4,28]. Of note, more than half of the cases were asymptomatic, which supports the need to perform lumbar punctures for an appropriate identification of CNS infiltration [29]. Seizures and headache were very prevalent symptoms in the group with NM, mainly during induction and re-induction therapy phases [27,30], which is in accordance to a report by Anastasopoulou et al. [15] that claimed the phenomenon to be related to therapy-induced neurotoxicity [18,31]. Conversely, Öztürk et al. [4] detected most NMs during the consolidation phase; however, it was not considered as CNS infiltration.

In addition, seizures are frequently reported during AL treatments in several reports, whereas older pediatric patients are at increased risks for seizures [15]. On the other hand, seizures in the context of hemato-oncologic CNS infiltrations have been related to intracranial hemorrhage, cerebral leukostasis, thrombosis, cerebral edema, metabolic disturbs, drugs and acute neurotoxic reactions [13,16,17,21,29,30].

Other frequent NMs included neuropathies and stroke. The former are commonly related to medications such as vincristine and methotrexate [24,32], whereas hemorrhagic strokes may be due to coagulation disorders. Furthermore, thrombotic and hemorrhagic strokes may be related to L-asparaginase therapy, which induces an imbalance of pro- and anticoagulant factors. Interestingly, all cases thrombotic and hemorrhagic strokes in our study occurred after L-asparaginase administration [8,14,33,34].

Surprisingly, PRES did not show a higher prevalence in our cohort. Conversely, this NM is within the first places in other reports [35]. In this study, PRES may had been underdiagnosed because all children had a head CT scan but not all had Magnetic Resonance Imaging (MRI), which depended on the medical criteria of each assigned physician, which was different from the recommended MRI for all children with leukemia showing NM [25].

The leukemic infiltration of CNS may protect leukemic cells from chemotherapy [13], which is a major cause of treatment failure, and this is consistent with the trend of higher mortality observed in the group with NM or CNS infiltration [14]. Moreover, AML and MPAL seem to provide a worse prognosis of survival based on the time from NM to death. This information has been scarcely explored by other studies, and we suggest a potential effect from chemotherapy stages as well as subject-specific responses to treatments [36,37,38,39]. This is in accordance to the notion that patients with CNS infiltration retain a lower survival rate when compared to current success rates for AL therapy, mainly those with ALL, but MPAL may be underestimated [13]. This may suggest the clinical relevance of intentional screening for neurological findings.

Finally, the presence of NM was associated with an increased mortality risk [21,28,36]. We found a trend for lower mortality and longer time period from NM onset to death for the ALL group in comparison with other linages, which is consistent with the literature [40]. This observation is consistent with similar studies [41,42,43], although overall mortality was comparatively higher and mainly occurred during the first months after induction or re-induction phases, suggesting a therapy-adverse toxicity effect [14,28,31,39].

## 5. Conclusions

This study found that ALL was the most prevalent type of leukemia, whereas infiltration to CNS, seizures, headache, neuropathy and PRES was frequently observed NM. In general, NM was associated with a higher mortality rate, particularly during the induction or re-induction of therapy, possibly implying an adverse toxicity effect.

## Figures and Tables

**Table 1:** Distribution by age and sex in pediatric patients with AL.

	NM Group n = 106	Non-NM Group n = 501	Total n = 607	*p*-Value
	Mean (SD)			
Age of diagnosis of AL years	8.29 (4.9)	7.05 (4.5)	7.27 (4.5)	0.01
Sex				0.80
Female n (%)	49 (8)	225 (37)	274 (44.1)	
Male n (%)	57 (9.3)	276 (45.5)	333 (54.9)	
Age range				
<3 y n (%)	4 (3.8)	77 (15.4)	81 (13.3)	
3 y–5 y n (%)	15 (14.2)	187 (37.3)	202 (33.3)	<0.01
6 y–12 y n (%)	54 (50.9)	153 (30.5)	207 (34.1)	
12 y–18 y n (%)	33 (31.1)	84 (16.8)	117 (19.3	

Data are shown as frequencies and percentages. Abbreviations: NM, neurological manifestation; Non-NM, non-neurological manifestation. AL, acute leukemia.

**Table 2:** Classification of leukemia type by sex.

Hematological Diagnosis	Sex		Total	*p*-Value
	Boys	Girls		
ALL n (%)	286 (47.1)	222 (36.6)	508 (83.7)	<0.01
AML n (%) MPAL n (%)	39 (6.4) 8 (1.3)	52 (8.6) 0 (0.0)	91 (15.0) 8 (1.3)	
Total n (%)	333 (54.8)	274 (44.1)	607 (100.0)	

Data are shown as frequencies and percentages. Abbreviations: ALL, acute lymphoblastic leukemia; AML, acute myeloid leukemia; MPAL, mixed-phenotype acute leukemia.

**Table 3:** NM in the different phases of AL treatment.

	Induction	Consolidation	Maintenance	Reinduction	Vigilance	Palliative	Total	*p*-Value
Infiltration to CNS	0	0	0	28	0	0	28	≤0.0001
Seizure, no CT-toxicity suspected	8	2	3	1	3	0	17	
Seizure, CT-toxicity suspected	4	0	1	2	2	2	11	
Headache	5	4	5	2	4	0	20	
Neuropathy	1	4	6	1	0	0	12	
Alertness disorders	7	0	1	0	0	1	9	
Decreased visual acuity	1	2	0	0	0	0	3	
Facial palsy	2	0	0	0	0	0	2	
Cerebellar Syndrome	0	0	1	0	1	0	2	
Tremor	0	0	1	0	0	0	1	
Alterations of mental functions	0	0	0	1	0	0	1	
Total	28	12	18	35	10	3	106	

Data are shown as frequencies in the different phases of AL treatment. Abbreviations: CT, chemotherapy; AL, acute leukemia; CNS, central nervous system.

**Table 4:** Final neurological diagnosis.

Final Diagnosis	n (%)
Infiltration to CNS	59 (55.7)
Epilepsy	21 (15.9)
Primary Headache	18 (13.6)
First Generalized Seizure	9 (6.8)
Sensitive neuropathy	7 (5.3)
PRES	6 (4.5)
Post-puncture Headache	5 (3.8)
First Focal Seizure	4 (3)
Mix stroke	4 (3)
Ischemic stroke	4 (3)
Motor neuropathy	2 (1.5)
Hemorrhagic stroke	2 (1.5)
Transient stroke	2 (1.5)
Peripheral Facial Palsy	2 (1.5)
Tremor	1 (0.8)
Learning Disorder	1 (0.8)

Data are shown as frequencies and percentages. Abbreviations: CNS, central nervous system; PRES, posterior reversible encephalopathy syndrome.

## Data Availability

Datasets analyzed or generated during this study can be requested from the authors.
